# Evolving roles of scientists as change agents in science education over a decade: SFES roles beyond discipline-based education research

**DOI:** 10.1126/sciadv.aav6403

**Published:** 2019-06-05

**Authors:** Seth D. Bush, Michael T. Stevens, Kimberly D. Tanner, Kathy S. Williams

**Affiliations:** 1 California State Polytechnic University, San Luis Obispo, CA 93407, USA.; 2 Utah Valley University, Orem, UT 84058, USA.; 3 San Francisco State University, San Francisco, CA 94132, USA.; 4 San Diego State University, San Diego, CA 92182, USA.

## Abstract

To what extent have positions for science education specialists as change agents within science departments persisted and evolved over the past decade? We addressed this question by studying a population of Science Faculty with Education Specialties (SFES) first described in 2008. SFES are university science faculty who engage in undergraduate science education, K-12 science education, and/or research in science education. Compared to a decade ago, SFES are now more prevalent and more likely to be formally trained in science education. Many identify as discipline-based education researchers (DBER) but assert that their SFES and DBER roles are nonequivalent. SFES have garnered university administrator support through varied science education activities, and these insights into the evolving role of scientists in science education have implications for many stakeholders.

## Teaser

University science faculty with science education integral to their academic positions are more common than a decade ago.

## INTRODUCTION

The importance of effective science education is critical at this societal and political moment in the United States (*1*–*3*) and across the world (*4*, *5*). To solve complex problems like energy management, food insecurity, climate change, and disease epidemics, we need to succeed not only in scientific research but also in communicating these advances and their implications to broad audiences. Hence, all scientists are increasingly being asked to be ambassadors of science and expand their efforts to communicate their work broadly—in their university teaching, in partnerships with K-12 educators, on Twitter, with the press, and beyond. Yet, it is still the case that few scientists receive formal training in their own education about how to effectively teach and communicate what they know to others (*6*, *7*).

One institutional approach to addressing the need for improved and expanded efforts in science education has been to embed scientists who have specialized roles in science education—in undergraduate science education, K-12 science education, and/or science education research—squarely within science departments. In the previous decade, descriptions of these “Science Faculty with Education Specialties” (SFES) were published, demonstrating that natural science departments—for reasons unknown at the time—were positioning Ph.D.-level faculty in their midst to focus professional efforts on science education in the context of their departments (*8*–*14*). Subsequent national studies of SFES demonstrated that they were present in science departments in all institution types examined: Ph.D.-granting, M.S.-granting, and primarily undergraduate institutions (*11*). Most SFES reported their primary impact as influencing the teaching practices of fellow scientists within their institution, reaching beyond their own classrooms (*13*). In addition, SFES reported scholarly efforts in both K-12 science education and science education research, resulting in contributions with impacts beyond their institution (*13*). However, it is unclear whether the SFES phenomenon is transient or whether integration of science education expertise into the fabric of science departments and disciplines is becoming the norm. In addition, it is unclear what characterizes these positions, how they are viewed by administrators, and whether SFES efforts are synonymous with discipline-based education research (DBER) efforts in their field.

The overarching research question for this study was “To what extent have positions for science education specialists as change agents within science departments persisted and evolved over the past decade?” To address this research question, we took three approaches. First, we aspired to compare the current state of SFES to that in the original study of SFES in 2007, including analyses of SFES professional training, whether they were hired into their SFES positions (H-SFES) or had transitioned from an existing traditional faculty role (T-SFES), their perceptions about whether their work was understood and/or valued by others, their professional satisfaction, and the general demographics of the SFES respondent population (*9*). In addition, we aimed to measure other SFES characteristics that were not probed in the original 2007 study but have been studied among SFES nationally (*11*), such as perceptions about their arenas of impact and the origins of their current position, as well as their success in obtaining funding and fostering educational changes in their department. Last, we sought to initiate new lines of inquiry about how administrators view SFES positions, and how SFES training and professional efforts relate to DBER.

Here, we provide evidence that the SFES phenomenon has persisted, expanded, and evolved over the past decade. To match the context and methods originally used to describe the SFES phenomenon in 2007 (*9*), we collected these 10-year follow-up data in the context of the 23-campus California State University (CSU) system, the largest university system in the United States, which includes Ph.D.-granting, M.S.-granting, and primarily undergraduate institutions. Evidence about the current state of the SFES phenomenon was collected via online surveys (see the Supplementary Materials for survey instrument; appendix S1) from SFES, as well as via interviews with deans of Colleges of Science and/or Engineering. Where possible, we compared the current state of SFES to that originally studied in 2007 and published in 2008 (*9*). In addition, we invited reflections from SFES who had been in their positions since before 2007 about their perceptions of changes over the past decade. We compared characteristics of these long-standing SFES to SFES new to their positions since 2007. While many characteristics of SFES positions appear unchanged since their original description (*9*), SFES have persisted, shifted in training and funding profiles, garnered positive views by administrators, and emerged as nonsynonymous with DBER.

## RESULTS

### Increased number of SFES hired in natural science departments in the past decade

**Fig. 1:**
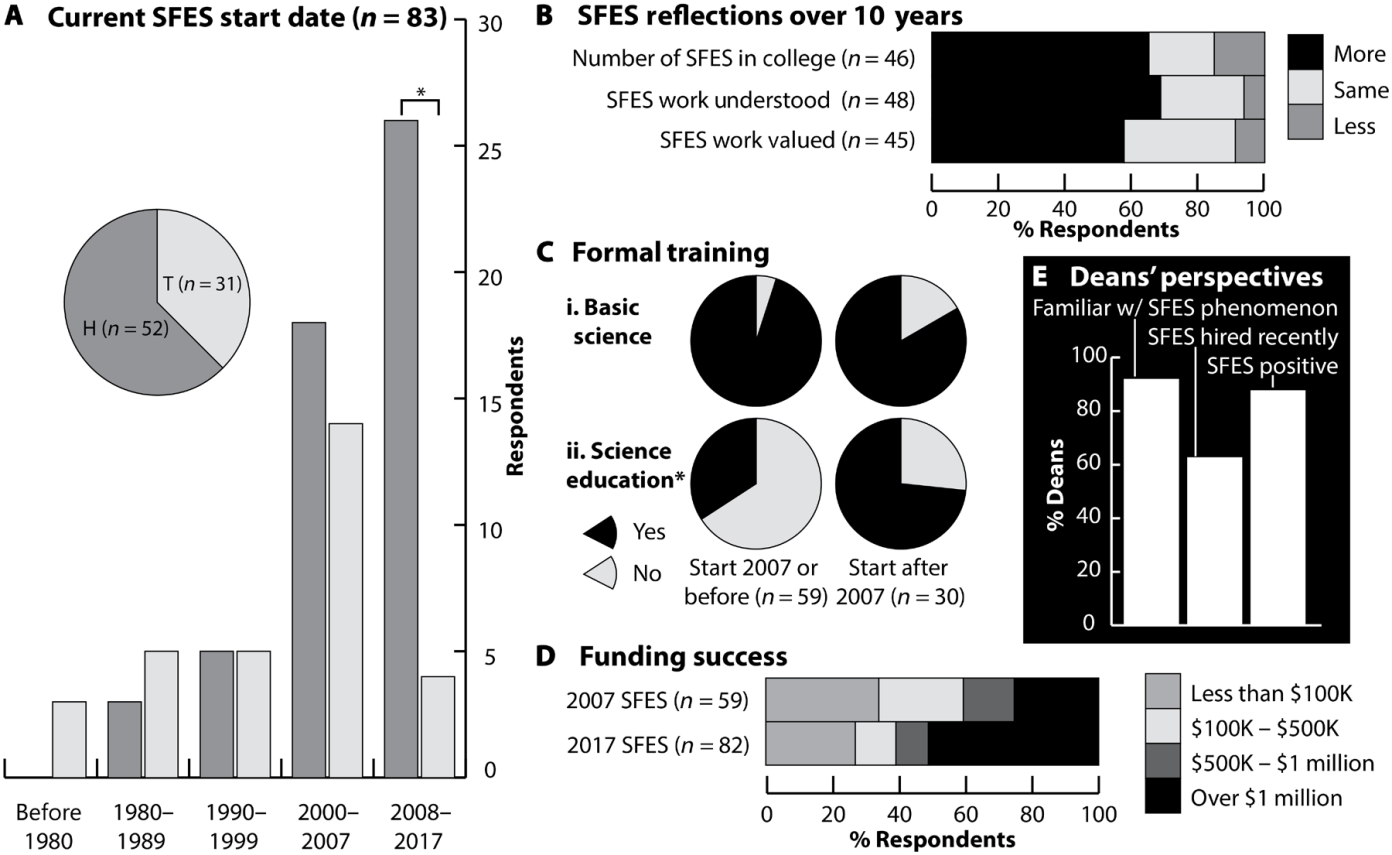
Evolution of SFES recognition, training, and funding. (**A**) SFES start dates disaggregated by those hired directly (H) and those who transitioned (T) into their SFES position. Pie inset describes proportions of H-SFES and T-SFES. *In the past decade, there were significantly more H-SFES than T-SFES (Pearson χ^2^ = 11.6, df = 1, *P* < 0.001). (**B**) Changes in perspectives on the SFES phenomenon reported by SFES who have been in their position for 10 or more years. (**C**) Reported formal training in (i) basic science and (ii) science education disaggregated by start date, 2007 or before (left) and after 2007 (right). *Those with start dates after 2007 were significantly more likely to report having formal training in science education (Pearson χ^2^ = 12.4, df = 1, *P* < 0.001). (**D**) Total funding reported for SFES in 2007 (top) and in 2017 (bottom). (**E**) Deans’ reported perspectives on the SFES phenomenon, recent SFES hiring, and attitudes toward SFES (*n* = 24).

Comparing the state of SFES in 2007 and 2017, the SFES phenomenon appears to have persisted and expanded, with a 51% increase in the absolute number of SFES respondents, from 59 in 2007 to 89 in the current study. In particular, a key shift over the past decade is that more SFES are being hired specifically into their position (H-SFES), with a concomitant decline in the number of SFES transitioning into these roles from existing faculty positions [T-SFES; *P* < 0.001; Fig. 1A; (*9*)]. Of those SFES in their position for ≥10 years, the majority asserted the presence of more SFES in their college, as well as more understanding and valuing of SFES work by their colleagues (Fig. 1B). These conclusions that SFES roles have persisted and increased are also supported by administrator perspectives on SFES hiring and impact (see below and Fig. 1E).

### Changes in SFES professional training and funding profile

More than twice as many SFES who started since 2007 reported formal training in science education compared to those who started before 2007, suggesting that training pathways for this career have emerged in the past decade (*P* < 0.001; Fig. 1C). In addition, more than half of 2017 SFES respondents reported obtaining ≥$1 million in grant monies to support their science education efforts compared to less than a quarter of 2007 SFES respondents (Fig. 1D). While one might associate this increase in funding with the increase in formal science education training, previous national studies of SFES have shown a disconnect, with no correlation between formal science education training and funding success (*11*). However, increases in SFES success in obtaining grant monies to support their science education efforts may be key to their integration into science departments. The ability of SFES to generate grant monies within their own discipline may legitimize them in their fields and contribute to their increased acceptance into science departments and colleges (see below).

### Local change agents: Insights into SFES impacts, origins, and identity

**Fig. 2:**
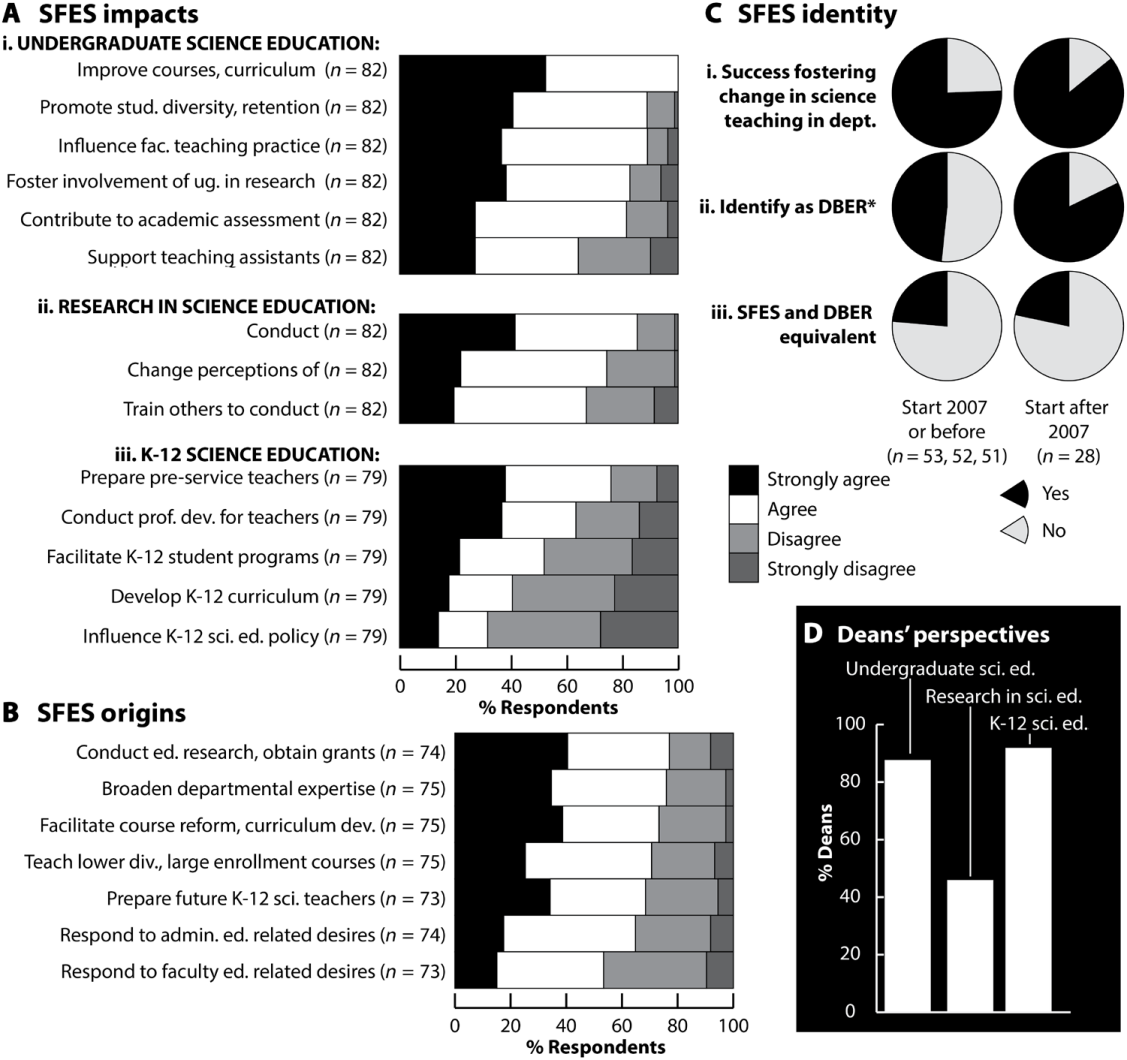
Change agency. (**A**) SFES reported impacts in three arenas of science education: (i) undergraduate science education, (ii) research in science education, and (iii) K-12 science education. (**B**) SFES perceptions of departmental motivations for creating SFES positions. (**C**) Elements of SFES identity including (i) success as a change agent, (ii) self-identity as a DBER, and (iii) lack of SFES and DBER equivalency, disaggregated by start date, 2007 or before (left) and after 2007 (right). *Those with start dates after 2007 were significantly more likely to identify as a DBER (Pearson χ^2^ = 8.8, df = 1, *P* = 0.003). (**D**) Deans’ perspectives of SFES activity/impacts in science education (*n* = 24).

In probing SFES perceptions of their impact, every SFES respondent reported improving courses and curriculum as a key impact (Fig. 2A). In addition, the majority of SFES endorsed every undergraduate science education impact probed, asserted impacts in conducting and changing perceptions of science education research, and reported impacts in preparing pre-service K-12 teachers (Fig. 2A). Most SFES continue to be engaged in multiple arenas of science education— undergraduate science education, K-12 science education, and science education research (Fig. 2D). In examining the origins of their position, SFES reaffirmed several previously reported departmental motivations (*12*, *14*) that are similar to their impacts and spanned conducting education research, obtaining grant monies, facilitating undergraduate education reform, and preparing future teachers (Fig. 2B). Many SFES additionally reported that their positions were driven by education-related aspirations of administrators and/or departmental faculty (Fig. 2B).

### Administrator perspectives on the SFES phenomenon

Administrator interviews (representing 22 of 23 CSU campuses) independently confirmed SFES expansion over the past decade, with >90% of interviewed deans reporting familiarity with SFES and 60% having recently hired an SFES (Fig. 1E). Consistent with SFES reports of increased understanding and valuing of their work by colleagues, the vast majority of deans (21 of 24; 87.5%) asserted positive impressions of SFES and their impact on their campuses. However, there were misalignments between SFES perceptions of impact and administrator perspectives on SFES impact (Fig. 2, A and D). While the majority in both groups assert a variety of SFES impacts in advancing undergraduate science education efforts, there appeared to be a disconnect in perceptions about SFES impact in the realms of K-12 science education and research in science education. More administrators asserted SFES impact on K-12 science education (22 of 24; 92%) than SFES impact through research in science education (13 of 24, 54%; Fig. 2D), whereas more SFES assert impact through research in science education (Fig. 2A, ii) than impact in K-12 science education (Fig. 2A, iii). These current findings echo previous published research on misalignments between SFES perceptions of the reasons for their hiring and their perceptions about their actual contributions (*12*). These misalignments have been hypothesized as one reason that ~40% of SFES in the original 2007 study and ~30% of SFES in a subsequent study of SFES across the United States were seriously considering leaving their position but not the field (*9*, *11*). Similar to previously published evidence, a relatively large proportion of SFES in the current study (29%) reported seriously considering leaving, with significantly more (91%) considering leaving their position and only 13% considering leaving the field (χ^2^ = 16.1, *P* < 0.001, McNemar’s test). Achieving clarity about SFES roles, expectations, and impacts appears to have been a persistent issue over the past decade.

### SFES and DBER are not equivalent

An emergent finding from this 10-year follow-up research is that an SFES role is seen as distinct from a DBER role by both faculty respondents (Fig. 2C, iii) and administrators (Fig. 2D). Note that many faculty respondents embraced both of these professional identities, as evidenced by their participation as SFES in this research study, as well as by their majority agreement that they identify as DBER (Fig. 2C, ii). However, while these professional identities may be simultaneously held by many individuals, they are not seen as equivalent, but rather representing different aspects of professional work (Fig. 2C, iii). The majority of current respondents rejected the equivalency of SFES and DBER (Fig. 2C, ii), as in the following examples:

“I think SFES encompasses a broader range of activities than DBER. For me, DBER means having a research program focused on improving science education, whereas SFES may include some DBER activities, but it may also include non-research activities, such as curriculum development, TA training & mentorship, K-12 teacher development, etc.”

“I see my work and the purpose [of] SFES as different because it goes beyond just [a] discipline-based education researcher. As your own categories have identified, it is research, but also influencing teaching, curriculum, and K-12 education. For me it goes beyond this still because much of my work is community-based and informal, so it also involves non-traditional partners in educational practice, evaluation, and shared learning. DBER to me implies that the focus is just on one of these areas, which is researching the education within your specific discipline.”

“I consider DBER to be a subset of SFES. I consider myself to be both. Most of my publications are highly discipline specific but I also do work that integrates science education scholarship/teaching/service.”

Similar to the sample responses above, we anticipate that SFES professional activities in the realms of undergraduate science education and K-12 science education including pedagogical professional development, course reform, program assessment, training of graduate student teaching assistants and pre-service teachers, and more are those that go beyond DBER activities, which appear to be perceived as solely research efforts. Given administrators’ perceptions of the key impacts of SFES (Fig. 2D), it appears that non-DBER activities are those with the greatest potential for institutional impact from SFES hiring. This is supported by previous research findings where SFES themselves reported influencing the teaching practices of departmental colleagues and fostering change from within departments as their biggest impact (*13*). Evidence from this study also supports this notion, as the vast majority of current SFES respondents asserted success in fostering change in science teaching in their department (Fig. 2C, i).

## DISCUSSION

### Implications for the changing role of scientists in science education

The evidence presented here supports the assertion that science education has increasingly become an area of professional work integrated into the natural sciences. This integration is being accomplished by SFES who are hired by science faculty colleagues and administrators who see the need for change agents in their departments. As the SFES phenomenon has become more accepted and understood, additional science education training pathways appear to have emerged to prepare scientists to address science education needs. Over the past decade, the SFES phenomenon has endured, expanded, and been embraced by administrators, especially for their role in undergraduate science education and K-12 science education. Given that previous research has shown that SFES perceive their largest impact to be their influence on the teaching practices of departmental colleagues, SFES are well positioned as a growing force in STEM (science, technology, engineering, mathematics) higher education reform, poised to foster educational change from within science departments (*13*). Last, although many SFES identify as DBER, they assert that SFES and DBER are not equivalent. Instead, SFES highlight their involvement in a wide range of activities including undergraduate science education and K-12 science education, in addition to research in science education. Therefore, if change and reform in science education is a priority, then a greater focus on hiring and supporting SFES is warranted, because SFES roles appear to reach beyond research-focused efforts of DBER.

### Limitations and considerations

These findings describe faculty occupying SFES roles in the CSU system. Although this sample represents a particular university system, the CSU is the largest university system in the United States with 23 campuses that include a variety of institution types. Furthermore, previous results from the CSU reflected the profile of SFES at institutions of higher education across the United States (*11*). This suggests that while many of the results presented here may represent the general SFES phenomenon, others may be unique to the CSU system. Increased awareness and visibility of the SFES phenomenon may have affected the size of the 2017 sample pool relative to 2007. As a result, perceived growth of CSU SFES, as evidenced by a larger apparent SFES population, deans’ perspectives regarding SFES hires (Fig. 1E), and 10-year CSU SFES reflections of SFES growth (Fig. 1B), may be an artifact of underreporting in 2007. Conducting a similar follow-up study of SFES across the United States could certainly provide additional insights.

## MATERIALS AND METHODS

### Sampling and data collection

#### CSU SFES survey

SFES in CSU science departments were identified for this study by (i) returning to the invitation list for the original 2007 CSU SFES investigation, (ii) examining all CSU science department websites for faculty profiles, and (iii) prompting initial survey respondents to provide names of additional CSU SFES. These data were collected 10 years after the original investigation of the SFES phenomenon in the CSU (*9*). This study was approved by California Polytechnic State University, San Luis Obispo’s Institutional Review Board (IRB). The subjects provided informed consent before participation following a procedure approved by the aforementioned IRB.

A total of 157 CSU faculty were invited to complete a 73-question anonymous online survey, which had been evaluated for face validity using non-CSU faculty, and 68 of the invitees responded to the survey between September 2017 and November 2017 (43% response rate). To increase participation, we sent a second invitation to initial nonrespondents and potential SFES named by initial survey respondents (*n* = 99). This second invitation included an offer of a $50 gift card incentive. All CSU SFES respondents were compensated, whether they responded before or after the offering of the incentive. An additional 42 of the invitees responded to the second survey between December 2017 and January 2018, which yielded a final overall 110 respondents for the study (66% response rate). Research participants were from 22 of 23 campuses, suggesting that our SFES census was comprehensive. Overall, 63% of SFES report having an SFES colleague in their department and 89% report having an SFES colleague in their college. Surveys with consent but no responses (*n* = 3), no consent (*n* = 2), or from individuals that did not identify as SFES (*n* = 16) were excluded. Of the remaining 89 survey respondents, all individuals self-identified as SFES.

**Fig. 3:**
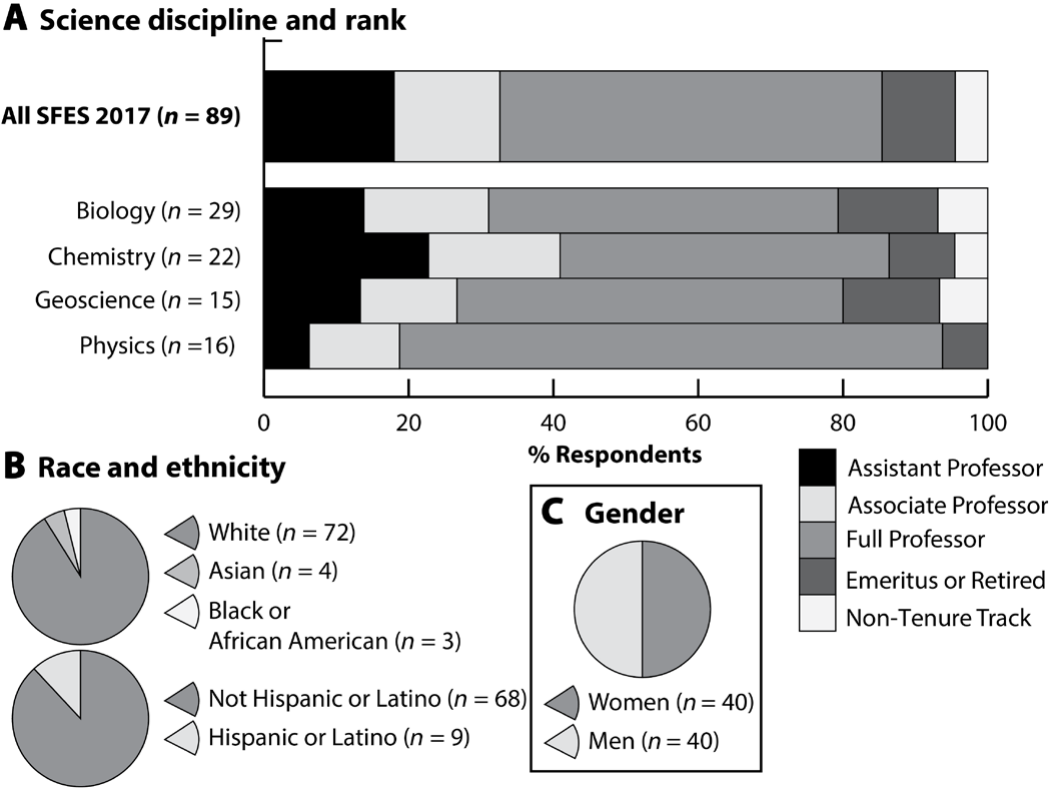
Demographics of SFES sample. (**A**) SFES disaggregated by discipline and rank. (**B**) SFES disaggregated by reported race and ethnicity. (**C**) SFES disaggregated by reported gender.

Because a substantial number of SFES respondents in the current survey could also have been sampled in 2007 (66% started in or before 2007; Fig. 1C), direct comparisons of the two datasets would have resulted in double counting of substantial numbers of individual SFES. In addition, the anonymity assured respondents in both the 2007 and the 2017 studies prevented a direct, longitudinal comparison approach. Hence, we used several analysis approaches to address our overarching research question about the persistence and evolution of SFES over the past decade. First, we presented results from all respondents in the current 2017 SFES study (e.g., Figs. 1A, 2, A and B, and 3). Second, we reported reflections on changes in the SFES phenomenon over the past decade from only those individuals who had been in their SFES positions over the past 10 years (e.g., Fig. 1B). Third, we made comparisons within the current 2017 dataset between those SFES who started in or before 2007 and those SFES who started after 2007 (e.g., Figs. 1C and 2C). Last, we did make some direct comparisons between the original 2007 dataset and the current 2017 dataset (e.g., Fig. 1D, Funding success).

These SFES represented four science disciplines, including biology (33%), chemistry (25%), geoscience (17%), and physics (18%), as well as science faculty in centers for science and math education housed in science colleges (8%; Fig. 3). Eighty-one percent identified as white held positions across all faculty ranks (4% Non-Tenure Track, 18% Assistant Professor, 15% Associate Professor, 53% Full Professor, and 10% Emeritus) and were trained extensively as researchers in basic science. Equal numbers (*n* = 40; 50%) characterized themselves with gender identities of woman or man (Fig. 3). These descriptive characteristics of SFES in the current study were not significantly different from the original study a decade ago (*9*).

#### CSU dean interviews

In June 2017, we contacted by email the current dean or current interim dean of each CSU if they had been in their position for at least 2 years. If the current dean or current interim dean had served for fewer than 2 years, we contacted his/her closest predecessor who had served as dean or interim dean for at least 2 years. In our initial contact, we described the nature of our study and asked them to provide informed consent, their telephone contact information, and the best times to be interviewed. In two cases, a dean selected using this protocol, but who had served a relatively short time, strongly encouraged us to interview a previous or current dean who they felt had critical experience with and knowledge of the SFES situation and its history at that campus. In those two cases, we interviewed a second dean who helped complete responses to our research questions about the situation of SFES on their campus over the past 10 years. Twenty-four deans from 22 of 23 CSU campuses completed an interview (96% campus response rate). Interviews were completed between July 2017 and January 2018.

Subjects were scheduled for 30-min telephone interviews using the contact information provided. As part of their interview-scheduling email, each participant was given our research goals, the categories of questions that we would ask, the identities of the researchers who would conduct the interview, and assurances of confidentiality.

Interviews were conducted and audio-recorded by two interviewers, one of whom took a more active role and asked the majority of questions, while the second interviewer was present to ensure consistency in the interview protocol and to provide a backup recording. During the interview, participants were addressed using their actual name, but during transcription and analysis, pseudonyms replaced actual names, and institution names were redacted to protect the identities of our participants. All participants agreed to be recorded and to have their interview transcribed. We provided a copy of their interview transcript to participants who requested it.

Our semistructured interviews were conducted using a protocol that included an informational preamble followed by five main questions centered on (i) their current awareness of SFES, (ii) the current state of SFES on their CSU campus, (iii) the motivations for the creation of SFES positions on their CSU campus, (iv) their perceptions of changes in the SFES phenomenon over that past 10 years at their CSU campus, and (v) any other ideas about SFES that they would like to share.

### Statistical analysis

Pearson χ^2^ tests of independence were used to assess whether paired observations were independent of each other [e.g., responses of those hired directly into their SFES position (H-SFES) versus those who transitioned into their SFES position (T-SFES), or SFES with start dates in or before 2007 versus those who started after 2007]. McNemar’s test was used to compare paired proportions, such as comparing SFES who were “seriously considering leaving” their “position” or “field.” A χ^2^ probability of 0.05 or less was used to justify rejecting the null hypothesis that the values from two subpopulations of SFES were unrelated to each other.

## Supplementary Material

http://advances.sciencemag.org/cgi/content/full/5/6/eaav6403/DC1

Download PDF

## SUPPLEMENTARY MATERIALS

Supplementary material for this article is available at http://advances.sciencemag.org/cgi/content/full/5/6/eaav6403/DC1 Appendix S1. SFES survey.
