# Supplementary material for: Evolving roles of scientists as change agents in science education over a decade: SFES roles beyond discipline-based education research
Source: Sci Adv. 2019 Jun 5;5(6):eaav6403. doi: 10.1126/sciadv.aav6403 (PMC6551186; doi:10.1126/sciadv.aav6403)
Supplement: http://advances.sciencemag.org/cgi/content/full/5/6/eaav6403/DC1 [file supp_5_6_eaav6403__index.html]

Science Advances | Science Advances

## Supplementary Materials

**This PDF file includes:**

- Appendix S1. SFES survey.

Download PDF

**Files in this Data Supplement:**

- Adobe PDF - aav6403\_SM.pdf
