# Supplementary material for: Evolving roles of scientists as change agents in science education over a decade: SFES roles beyond discipline-based education research
Source: Sci Adv. 2019 Jun 5;5(6):eaav6403. doi: 10.1126/sciadv.aav6403 (PMC6551186; doi:10.1126/sciadv.aav6403)
Supplement: Download PDF [file aav6403_SM.pdf]

Science Advances
AAAS

advances.sciencemag.org/cgi/content/full/5/6/eaav6403/DC1

Supplementary Materials for

Evolving roles of scientists as change agents in science education over a decade: SFES roles beyond discipline-based education research

Seth D. Bush, Michael T. Stevens, Kimberly D. Tanner, Kathy S. Williams*

*Corresponding author. Email: kathy.williams@sdsu.edu

Published 5 June 2019, *Sci. Adv.* **5**, eaav6403 (2019)
DOI: 10.1126/sciadv.aav6403

This PDF file includes:

Appendix S1. SFES survey.

Appendix S1. SFES survey.

Informed Consent Letter

Dear Colleague,

You are being asked to participate in a research study conducted by Dr. Seth Bush from the Department of Chemistry & Biochemistry at Cal Poly San Luis Obispo. Dr. Bush is part of a cross-University research team that includes: Dr. Michael Stevens at Utah Valley University, Dr. Kimberly Tanner at San Francisco State University, and Dr. Kathy Williams at San Diego State University. You were selected as a possible participant in this study because you were identified as a university science faculty member (tenured, tenure-track, or non-tenure-track) who may have specialized science education responsibilities beyond those of typical science faculty on your campus. Your participation in this study is voluntary.

PURPOSE OF THE STUDY: The purpose of this study is to investigate the characteristics, experiences, and responsibilities of Science Faculty with Education Specialties (SFES) in the California State University system and to document how these have evolved over the last ten years.

PROCEDURES: You will be asked to complete an on-line survey that asks you questions about your current professional position and professional activities, as well as your perceptions about issues related to your position. If you volunteer to participate in this study, you will complete the survey anonymously using a secure website. You will be giving us permission to read, analyze, and report data resulting from your anonymous responses to the survey. The survey should take approximately 30 minutes to complete.

POTENTIAL RISKS AND DISCOMFORTS: You are unlikely to be exposed to any potential risks or discomforts by participating in this study.

POTENTIAL BENEFITS TO YOU AND/OR SOCIETY: There may be some direct benefits to you by participating in this study. You may find the survey enhances your awareness of one or more issues that impact your professional success. Reported findings may include information that would have the potential for improving your administrative support, financial support, and career opportunities. Higher education, and science education in particular, will potentially benefit from an investigation of the experiences of this group of faculty. University administrators, faculty candidates, and faculty hires will have data that may enhance hiring and retention success for this type of position.

PAYMENT FOR PARTICIPATION: You will not be paid for participating in this research project.

CONFIDENTIALITY AND ANONYMITY: Your identity will be kept strictly confidential during the entire research process, and the survey data itself will be collected anonymously. We are interested in the set of responses as a whole, not a particular individual's responses. Anonymous data or findings from this study might be included in various publications or presentations. The survey data will be stored in a secure, locked location for up to five years from the collection date.

PARTICIPATION AND WITHDRAWAL: You can choose whether to participate, and you may withdraw from the study at any time. Choosing not to participate or choosing to withdraw at any point will mean that your responses will not be included in data analysis or reporting for research purposes. You also do not have to answer any questions you choose not to.

IDENTIFICATION OF INVESTIGATORS: If you have any questions or concerns about the study, please feel free to contact Dr. Bush, who can be reached at (805) 756-2746 or by email at sbush@calpoly.edu.

RIGHTS OF RESEARCH PARTICIPANTS: You may withdraw your consent at any time and discontinue participation without penalty. If you have questions or concerns regarding the manner in which the study is conducted, you may contact Dr. Michael Black, Chair of the Cal Poly Institutional Review Board, at (805) 756-2894, mblack@calpoly.edu, or Dr. Dean Wendt, Dean of Research, at (805) 756-1508, dwendt@calpoly.edu.

Thank you in advance.

* 1. I have read the procedures described above. By checking “Agree” below, I am electronically signing this document and consenting to participate in this study.

◯ Agree

◯ Disagree

Overview ...

While all college and university science faculty are education specialists in some regard through their teaching responsibilities, here we define Science Faculty with Education Specialties (SFES) as faculty either:

1) who have been specifically hired in science departments to specialize in science education beyond typical faculty teaching duties.
OR
2) who have transitioned after their initial hire to a role as a faculty member focused on issues in science education beyond typical faculty teaching duties.

This study of Science Faculty with Education Specialties (SFES) in the California State University system intends to collect descriptive information about SFES across different science disciplines and across different types of higher education institutions. In addition, the study also aspires to collect evidence from university science faculty who occupy a variety of positions – not just tenured/tenure-track positions – and who are engaged in a variety of activities related to science education.

To capture the characteristics of this varied population, the survey contains the following nine sections:

1. On Your Position ...
2. About Your Teaching Activities ...
3. About Your Scholarly Activities ...
4. About Your Service Activities ...
5. About Your Professional Training ...
6. About Your Professional Satisfaction ...
7. About Your Specific SFES Position ...
8. About You ...
9. For the Future ...

At the beginning of each section, there will be an introductory page that gives an overview of the content of the section.

As SFES ourselves, we have attempted to streamline the survey to minimize the time required for its completion. That said, we have endeavored to create a survey that will enable you to fully describe your situation, with ample opportunity to provide optional, open-ended comments. You should expect to spend about 30 minutes completing this survey. To minimize potential technical difficulties in completing the survey, we encourage you to complete it in one sitting if at all possible. If this is not possible, please continue to use the same computer and the same browser, so that you can return to your same survey in progress.

While we have designed this survey to capture the SFES experience for individuals across disciplines and types of institutions, we acknowledge that there may be places where you are unable to fully express the nature of your current professional position. Please use the comment boxes at the end of each section and at the end of the survey to share additional information about your situation that you feel is important. It may be helpful to have a copy of your CV handy.

Thank you in advance for your time and thoughtfulness in completing the survey.

On Your Position ...

On Your Position ...

In this section of the survey, you will be asked about whether you consider yourself an SFES, the characteristics of your current academic position, and how others in your institution may perceive your position.

On Your Position ...

Based on our definition of SFES as faculty either:
1) who have been specifically hired in science departments to specialize in science education beyond typical faculty teaching duties.

OR

2) who have transitioned after their initial hire to a role as a faculty member focused on issues in science education beyond typical faculty teaching duties.

2. Do you consider yourself to be a Science Faculty with an Education Specialty (SFES), as described above?

- ◯ Yes
- ◯ No, I don't consider myself to be an SFES because ...

On Your Position ...

3. Which of the following best describes your current position as a Science Faculty with an Education Specialty (SFES)?

- ◯ I was HIRED into a science faculty position, specifically to specialize in science education beyond typical faculty teaching duties
- ◯ I TRANSITIONED into a specialized science education role, beyond typical faculty teaching duties, after being hired into a science faculty position
- ◯ I am not sure either of these choices best describes my position because ... (please comment)

On Your Position ...

4. What year were you hired into your current position?

5. At what rank were you hired in your current position?

If Other, please elaborate.

6. What is your current rank?

If Other, please elaborate.

7. Which best describes your tenure status?

- I am Tenured
- I am in a Tenure-Track position, but am currently not Tenured
- I am in a Non-Tenure-Track position

8. Did you have tenure before adopting your role as an SFES?

- Yes
- No
- Not applicable because I am in a Non-Tenure-Track position

On Your Position ...

9. What is the full name of the SCIENCE DEPARTMENT that houses your position?

10. Which field designation best reflects the SCIENCE DEPARTMENT that houses your current position?

- Biology
- Chemistry
- Geoscience
- Physics
- Other

If Other, please elaborate.

11. Were you hired 100% into this SCIENCE DEPARTMENT?

- Yes
- No. Briefly describe how your appointment is structured.

On Your Position ...

12. Which best describes your INSTITUTION?

◯ Primarily Undergraduate ◯ Master's-degree granting ◯ Ph.D.-granting

13. What is the highest degree offered through your _____________?

	Bachelor's	Master's	Doctorate
Department	◯	◯	◯
College	◯	◯	◯

14. Which CAMPUS houses your current position?

[ ]

15. Which, if any, of these prior connections do you have to your INSTITUTION? Mark all that apply.

- [ ] You are an ALUMNA/US of your institution.
- [ ] You held a PREVIOUS JOB, different in kind, at your institution.
- [ ] You had a SPOUSE or PARTNER who worked or had accepted a position at your institution.
- [ ] You had NO prior connection (academic, professional, or spousal) to your institution.
- [ ] OTHER prior connection (please describe)

On Your Position ...

16. Not including yourself, does your department or college currently have any other Science Faculty with Education Specialties?

	Yes	No
Department	◯	◯
College	◯	◯

If yes, please list the names of these individuals. Your name will not be associated with this list, and this list, like all of your survey responses, will be kept strictly confidential.

17. Have you been in your current SFES position for 10 or more years?

- Yes
- No

On Your Position ...

18. Consider the last 10 years.

	More	Less	No more or less	Not Sure
There are ______SFES in my DEPARTMENT today than there were 10 years ago.	◯	◯	◯	◯
There are ______ SFES in my COLLEGE today than there were 10 years ago.	◯	◯	◯	◯
The work of SFES on my campus is ______ likely to be UNDERSTOOD by my colleagues today than it was 10 years ago.	◯	◯	◯	◯
The work of SFES on my campus is ______ likely to be VALUED by my non-SFES colleagues today than it was 10 years ago.	◯	◯	◯	◯
The work of SFES on my campus is ______ SUPPORTED by my non-SFES colleagues today than it was 10 years ago.	◯	◯	◯	◯
SFES on my campus have______access to undergraduate or graduate researchers today than they had 10 years ago.	◯	◯	◯	◯

	More	Less	No more or less	Not Sure
The retention/tenure/ promotion process for SFES on my campus is ______clear today than it was 10 years ago.	○	○	○	○
An SFES hired today __________likely to get tenure in my department than one hired 10 years ago.	○	○	○	○

On Your Position ...

Consider the last 10 years. Briefly address each prompt.

19. In which positions has there been a change in leadership in the last 10 years? Mark all that apply.

- ☐ Department Chair
- ☐ College Dean
- ☐ University Provost
- ☐ University President

20. How, if at all, has the situation for SFES on your campus changed in the last decade?

21. What are key elements that have contributed to the longevity of your role as an SFES?

About Your Teaching Activities ...

In this section of the survey, you will be asked about the TEACHING activities, responsibilities, and opportunities you have as an SFES. Please only consider the activities associated with your current position in answering the questions below.

About Your Teaching Activities ...

22. Compared to typical non-SFES faculty members in my department, I spend ________ of my time engaged in teaching activities.

◯ much more ◯ more ◯ about the same amount ◯ less ◯ much less ◯ no basis for judgment

Please feel free to comment on the amount of time you spend on teaching activities.

About Your Teaching Activities ...

23. Have you taught or are you currently teaching courses in the following categories? Please mark all that apply.

- ☐ Lower division majors core
- ☐ Upper division majors core
- ☐ Large enrollment courses
- ☐ Majors elective
- ☐ General education
- ☐ Graduate majors core
- ☐ Graduate majors elective
- ☐ Credential program courses including teaching methods courses
- ☐ Science courses specifically for future K-12 teachers

24. Please choose one of the following responses to indicate to what extent you agree with each statement.

	Strongly agree	Agree	Disagree	Strongly disagree	No basis for judgment
I am currently fulfilled by my teaching activities.	◯	◯	◯	◯	◯
I am teaching course(s) that I do not want to teach because I am an SFES.	◯	◯	◯	◯	◯

About Your Teaching Activities ...

25. My department has an UNDERGRADUATE curriculum for students interested in SCIENCE TEACHING on par with the curriculum for students interested in BASIC SCIENCE.

	Strongly agree	Agree	Disagree	Strongly disagree	No basis for judgment
Course(s)	○	○	○	○	○
Degree option(s)	○	○	○	○	○

26. My department has an UNDERGRADUATE curriculum for students interested in RESEARCH in SCIENCE EDUCATION on par with the curriculum for students interested in BASIC SCIENCE.

	Strongly agree	Agree	Disagree	Strongly disagree	No basis for judgment
Course(s)	○	○	○	○	○
Degree option(s)	○	○	○	○	○

27. My department has a GRADUATE curriculum for students interested in SCIENCE TEACHING on par with the curriculum for students interested in BASIC SCIENCE.

	Strongly agree	Agree	Disagree	Strongly disagree	No basis for judgment
Course(s)	○	○	○	○	○
Degree option(s)	○	○	○	○	○

28. My department has a GRADUATE curriculum for students interested in RESEARCH in SCIENCE EDUCATION on par with the curriculum for students interested in BASIC SCIENCE.

	Strongly agree	Agree	Disagree	Strongly disagree	No basis for judgment
Course(s)	○	○	○	○	○
Degree option(s)	○	○	○	○	○

About Your Teaching Activities ...

29. Please feel free to make additional comments about your teaching situation as an SFES.

About Your Scholarly Activities ...

In this section of the survey, you will be asked about the SCHOLARLY activities, responsibilities, and opportunities associated with your current academic position. Here, we define scholarly activities broadly. Below, you will be asked questions about four specific arenas of scholarly activities in which SFES may engage.

These arenas are:
1) Basic Science Research
2) Science Education Research
3) K-12 Science Education Activities
4) Undergraduate Science Education Activities

We realize that there will be some overlap in the four arenas delineated above. In addition, you will also be asked questions about your scholarly activities overall.

Please only consider the activities associated with your current position in answering the questions below.

About Your Scholarly Activities ...

30. Compared to typical non-SFES faculty members in my department, I spend _________ of my time engaged in scholarly activities.

○ much more ○ more ○ about the same amount ○ less ○ much less ○ no basis for judgment

Please feel free to comment on the amount of time you spend on scholarly activities.

About Your Scholarly Activities ...

In responding to the following questions, please consider the following descriptions:

Basic Science Research.
We ask that you consider basic science research to be research in the sciences that does not include science education.

Science Education Research.
We ask that you consider any research in science education including but not limited: research on issues of student conceptions, teaching and learning strategies, equity and diversity in the sciences, discipline-based science education issues, and the role of scientists in science education generally.

K-12 Science Education Activities.
We ask that you consider K-12 Science Education Activities as including but not limited to K-12 curriculum development, teacher preparation and professional development projects, and diversity and outreach projects.

Undergraduate Science Education Activities.
We ask that you consider Undergraduate Science Education Activities as including but not limited to curriculum development, instructional training for faculty or graduate teaching assistants, formal and informal faculty professional development in science teaching, and recruitment/retention outreach projects.

31. In your work as an SFES, how have you been involved in each of these arenas? Check all that apply.

	Basic Science Research	Science Education Research	K-12 Science Education Activities	Undergraduate Science Education Activities
Published articles in peer reviewed journals that relate to your scholarly activities	☐	☐	☐	☐
Applied for grants to support your scholarly activities	☐	☐	☐	☐
Presented results of your scholarly activities in this arena at regional, national, or international meetings	☐	☐	☐	☐

Other (please describe)

☐

About Your Scholarly Activities ...

32. To what extent do you agree with the following statements.

	Strongly agree	Agree	Disagree	Strongly disagree
My department is supportive of my scholarly activities.	○	○	○	○
I am currently fulfilled by my scholarly activities.	○	○	○	○
I am doing the scholarly activities that I thought I was hired to do.	○	○	○	○
I have the same academic freedom in developing research projects as my non-SFES peers.	○	○	○	○
I have adequate lab space to accomplish my scholarly activities.	○	○	○	○
I feel that my department has a culture supportive of RESEARCH in BASIC SCIENCE.	○	○	○	○
I feel that my department has a culture supportive of RESEARCH in SCIENCE EDUCATION.	○	○	○	○
I feel that my department has a culture supportive of ACTIVITIES in K-12 EDUCATION.	○	○	○	○
I feel that my department has a culture supportive of ACTIVITIES in UNDERGRADUATE SCIENCE EDUCATION.	○	○	○	○

About Your Scholarly Activities ...

33. In your current position...

	$0	$1 to $10K	$10K to $49K	$50K to $99K	$100K to $499K	$500K to $1 million	$1 million to $2 million	$2 million to $5 million	$5 million to $10 million	More than $10 million
How much grant money have you obtained in TOTAL?	○	○	○	○	○	○	○	○	○	○
How much grant money have you obtained to support BASIC SCIENCE RESEARCH?	○	○	○	○	○	○	○	○	○	○
How much grant money have you obtained to support SCIENCE EDUCATION RESEARCH?	○	○	○	○	○	○	○	○	○	○
How much grant money have you obtained to support K-12 SCIENCE EDUCATION?	○	○	○	○	○	○	○	○	○	○
How much grant money have you obtained to support UNDERGRADUATE SCIENCE EDUCATION?	○	○	○	○	○	○	○	○	○	○

About Your Scholarly Activities ...

34. The number of graduate student researchers available to participate in my scholarly activities is _________ the number available to participate in the scholarly activities of non-SFES faculty in my department.

○ much more than ○ more than ○ about the same as ○ less than ○ much less than

○ not applicable because my department does not have a graduate program

About Your Scholarly Activities ...

For the next two questions, you will be asked to reflect on your scholarly activities by answering the same questions we asked in our 2007 CSU study, to enable direct comparisons to that data set.

35. In your current position, what types of scholarly activities are you engaged in or have you engaged in?

Please mark all that apply.

- [ ] Research in basic science (not related to science education)
- [ ] Research in science education
- [ ] K-12 teacher development projects, e.g., recruitment, retention, professional development activities, etc.
- [ ] Curriculum development
- [ ] University teacher development projects, e.g., TA training, faculty workshops, etc.

Other (please specify)

36. In your current position, what types of projects have you applied for grant funding to support?

Please mark all that apply.

- [ ] Research in basic science (not related to science education)
- [ ] Research in science education
- [ ] K-12 teacher development projects, e.g., recruitment, retention, professional development activities, etc.
- [ ] Curriculum development
- [ ] University teacher development projects, e.g., TA training, faculty workshops, etc.

Other (please specify)

About Your Scholarly Activities ...

37. Please feel free to make additional comments about your scholarly activity situation as an SFES.

About Your Service Activities ...

In this section of the survey, you will be asked about the SERVICE activities, responsibilities, and opportunities associated with your current academic position.

Here, service activities are broadly defined as including but not limited to committee work, coordination duties, teacher preparation, evaluation and assessment, and other service outside of your institution.

Please only consider the activities associated with your current position in answering the questions below.

About Your Service Activities ...

38. Compared to typical non-SFES faculty members in my department, I spend ____________ of time engaged in service activities.

◯ much more ◯ more ◯ about the same amount ◯ less ◯ much less ◯ no basis for judgment

Please feel free to comment on the amount of time you spend on service.

About Your Service Activities ...

39. For each of the following service activities, consider your contributions and the contributions of a typical non-SFES peer in your department. Mark all that apply.

	I engage in this activity	My non-SFES peers engage in this activity
Service in my department(s)	☐	☐
Service in the science college (e.g., College of Science, College of Natural & Social Sciences, College of Science & Engineering, ...)	☐	☐
Service in the education college (e.g., College of Education)	☐	☐
Committee service at the university level	☐	☐
Coordination duties for courses that include supervision and/or training	☐	☐
Coordination duties for courses that include curriculum development	☐	☐
K-12 teacher preparation activities for pre-service teachers, including recruitment efforts	☐	☐
Activities for in-service K-12 teachers, such as professional development workshops	☐	☐
Evaluation or assessment activities, such as program review, curriculum assessment, etc.	☐	☐
Other service activities in your professional field outside of your institution, e.g., professional societies, grant agencies, etc. (please specify in the textbox below)	☐	☐

Other service activities or comments on your response (please specify)

About Your Service Activities ...

40. Please choose one of the following responses to indicate to what extent you agree with each statement.

	Strongly agree	Agree	Disagree	Strongly disagree	No basis for judgment
I am currently fulfilled by my service activities.	○	○	○	○	○
I am doing the service activities that I thought I was hired to do.	○	○	○	○	○

About Your Service Activities ...

41. Please feel free to make additional comments about your service situation as an SFES.

About Your Professional Training ...

In this section of the survey, you will be asked about your formal and informal professional training.

The first group of questions will ask you about your training in SCIENCE, while the second group of questions will ask you about your training in SCIENCE EDUCATION. Please keep this distinction in mind as you reply to the questions in this section.

About Your Professional Training ...

Consider your professional training in SCIENCE.

42. Please identify your FORMAL training in SCIENCE.

MARK ALL THAT APPLY, even if you have also done more advanced work.

- [ ] Postdoc in science field in a science department or college
- [ ] Postdoc in science field in a national laboratory
- [ ] Ph.D. in science field from a science department or college
- [ ] Masters Degree in science field from a science department or college
- [ ] Bachelors Degree in science field from a science department or college
- [ ] Minor in science field from a science department or college
- [ ] Course work in science field from a science department or college
- [ ] Graduate level research in science
- [ ] Undergraduate level research in science
- [ ] None of these apply

Other (please specify)

43. Please identify your INFORMAL or LESS FORMAL training in SCIENCE. MARK ALL THAT APPLY.

- [ ] Experience in private sector
- [ ] Experience in the public sector
- [ ] Sabbatical experience
- [ ] On the job experience
- [ ] Professional development workshops, seminars or short courses
- [ ] Self taught
- [ ] None of these apply

Other informal or less formal training (please elaborate)

About Your Professional Training ...

Consider your professional training in SCIENCE EDUCATION.

44. Please identify your FORMAL training in SCIENCE EDUCATION.

MARK ALL THAT APPLY, even if you have also done more advanced work.

- ☐ Postdoc in science education in a science or education department
- ☐ Ph.D. with an education emphasis from a science department
- ☐ Ph.D. or equivalent degree from an education department
- ☐ Masters in science education
- ☐ Graduate level research in science education
- ☐ Undergraduate level research in science education
- ☐ Minor in education
- ☐ K-12 teaching credential
- ☐ NSF GK-12 graduate fellowship
- ☐ NSF Postdoctoral Fellowship in Science, Math, Engineering, or Technology Education (PFSMETE)
- ☐ Other NSF sponsored graduate fellowship in science education
- ☐ Other NSF sponsored postdoctoral fellowship in science education
- ☐ None of these apply

Other formal training (including other NSF sponsored training) (please elaborate)

45. Please identify your INFORMAL or LESS FORMAL training in SCIENCE EDUCATION. MARK ALL THAT APPLY.

- [ ] Experience in private sector
- [ ] Experience in the public sector
- [ ] Sabbatical experience
- [ ] On the job experience
- [ ] K-12 teaching experience
- [ ] Professional development workshops, seminars or short courses
- [ ] Self taught
- [ ] TA Training program in graduate school
- [ ] Science education training programs in graduate school
- [ ] Leadership experience on science education project
- [ ] None of these apply.

Other informal or less formal training (please elaborate)

About Your Professional Satisfaction ...

This section will ask a wide range of questions that center on your professional satisfaction associated with your current academic position. A subset of questions will ask you to make comparisons between SFES and non-SFES in your department.

About Your Professional Satisfaction ...

46. Please choose one of the following responses to indicate to what extent you agree with this statement.

	Strongly agree	Agree	Disagree	Strongly disagree	Prefer not to answer
I am professionally satisfied in my current position.	○	○	○	○	○
I am doing the job I was hired to do.	○	○	○	○	○
SFES and non-SFES faculty in my department have similar job expectations.	○	○	○	○	○
I am confident that the retention/tenure/promotion review committees at my institution are qualified to review my accomplishments as an SFES.	○	○	○	○	○

If you are not confident in your retention/tenure/promotion review committees, what would give you greater confidence in the review process?

About Your Professional Satisfaction ...

47. Please choose one of the following responses for each statement.

	much more than	more than	about the same as	less than	much less than	no basis for judgment
My CURRENT salary is ___________the salaries of non-SFES peers in my department with similar years of service.	◯	◯	◯	◯	◯	◯
My STARTING salary was___________the salaries of non-SFES peers in my department with similar years of service.	◯	◯	◯	◯	◯	◯
My START-UP PACKAGE was ___________the start-up package of non-SFES peers in my department.	◯	◯	◯	◯	◯	◯

48. My lab space is ________________ the lab space of my non-SFES peers in my department.

◯ much bigger than ◯ bigger than ◯ about the same as ◯ smaller than ◯ much smaller than

◯ about the same because faculty in my department do not have lab space

About Your Professional Satisfaction ...

49. Briefly, what were your primary reasons for taking your current position?

50. Briefly, what are the primary reasons you continue to stay in your current position?

About Your Professional Satisfaction ...

51. I am seriously considering leaving my current_________________.

	Strongly agree	Agree	Disagree	Strongly disagree	Prefer not to answer
POSITION	◯	◯	◯	◯	◯
FIELD	◯	◯	◯	◯	◯
INSTITUTION	◯	◯	◯	◯	◯

If you are seriously considering leaving your POSITION, FIELD, or INSTITUTION, please provide more detail on your primary reason(s) for this consideration.

About Your Specific SFES Position ...

In this section of the survey, you will be asked questions about your CURRENT SFES position, your insights into the motivations for hiring someone into your position, and your perceptions of your impact in the position.

About Your Specific SFES Position ...

52. Below is a list of rationales SFES have offered to explain the motivation(s) for the creation and/or hiring of their SFES position. To what extent do you agree that each rationale reflects your understanding of the motivation(s) behind the creation of your CURRENT SFES position?

	Strongly agree	Agree	Disagree	Strongly disagree
To teach lower division and large enrollment courses.	○	○	○	○
To teach upper division courses.	○	○	○	○
To facilitate course reform or curriculum development.	○	○	○	○
To coordinate lab courses, teaching assistants, or other course support.	○	○	○	○
To prepare future K-12 science teachers.	○	○	○	○
To conduct educational research or obtain grant money.	○	○	○	○
To broaden departmental expertise.	○	○	○	○
To respond to administrative desires related to education.	○	○	○	○
To respond to departmental faculty desires related to education.	○	○	○	○
To replace a retiring or departing faculty member.	○	○	○	○
To relieve other faculty from teaching and service burdens.	○	○	○	○
Because SFES need only limited resources, such as space and start-up funds.	○	○	○	○
Because of my prior connection(s) to the institution.	○	○	○	○
I transitioned into this role, I was not hired as an SFES.	○	○	○	○

If these rationales do not reflect the motivations behind the creation and/or hiring of your position, please briefly share the motivations relevant to your position.

53. To the best of your knowledge, which of the following advocated for the creation and/or hiring of your current SFES position? Mark all that apply.

- ☐ Faculty in my department.
- ☐ Chair of my department.
- ☐ Dean of my college or other administrators in my college.
- ☐ Upper administration at my institution.
- ☐ People or groups external to my institution.
- ☐ I was not hired as an SFES.

About Your Specific SFES Position ...

54. Below is a list of impacts SFES have reported making in the area of RESEARCH IN SCIENCE EDUCATION. In my current SFES position, I have made impacts through ...

	Strongly agree	Agree	Disagree	Strongly disagree
Conducting research in science education.	○	○	○	○
Training others to conduct research in science education.	○	○	○	○
Changing perceptions of research in science education.	○	○	○	○

55. Below is a list of impacts other SFES have reported making in the area of K-12 SCIENCE EDUCATION. In my current SFES position, I have made impacts through ...

	Strongly agree	Agree	Disagree	Strongly disagree
Preparing pre-service teachers.	◯	◯	◯	◯
Conducting professional development for in-service teachers.	◯	◯	◯	◯
Facilitating programs for K-12 students.	◯	◯	◯	◯
Developing K-12 curriculum.	◯	◯	◯	◯
Influencing K-12 science education policy.	◯	◯	◯	◯

56. Below is a list of impacts other SFES have reported making in the area of UNDERGRADUATE SCIENCE EDUCATION. In my current SFES position, I have made impacts through ...

	Strongly agree	Agree	Disagree	Strongly disagree
Influencing faculty teaching practice.	◯	◯	◯	◯
Improving courses or curriculum.	◯	◯	◯	◯
Supporting teaching assistants.	◯	◯	◯	◯
Contributing to academic assessment.	◯	◯	◯	◯
Fostering involvement of undergraduates in research.	◯	◯	◯	◯
Promoting student diversity and retention.	◯	◯	◯	◯

57. Of all the impacts you have made as an SFES, what is the single MOST valuable impact YOU have made on your science department, college, and institution?

Department

College

Institution

About Your Specific SFES Position ...

58. Prior to my invitation to this study, I was familiar with the following terms...

	Yes	No
SFES: Science Faculty with Education Specialties	◯	◯
DBER: Discipline-based Education Researcher	◯	◯

59. I self-identify as a DBER: Discipline-based Education Researcher.

◯ Yes

◯ No

60. I consider the terms SFES and DBER to be equivalent to one another.

◯ Yes

◯ No

Please provide a brief explanation for your choice:

About Your Specific SFES Position ...

61. Please consider your current position and department.

	Yes	No
I aspire to foster change in how science is taught in my department.	◯	◯
I have had success in fostering change in how science is taught in my department.	◯	◯

62. Relative to the work of my non-SFES peers, I perceive that my SFES work is considered to be of ________________status by my...

	Higher	About equal	Lower
Non-SFES colleagues	◯	◯	◯
Departmental administrators	◯	◯	◯
College administrators	◯	◯	◯
University administrators	◯	◯	◯

About Your Specific SFES Position ...

63. Below is a list of advice other SFES have given for new SFES beginning in the field. Mark the extent to which you agree with this advice.

	Strongly agree	Agree	Disagree	Strongly disagree
Find colleagues, mentors, and advocates.	○	○	○	○
Obtain clear expectations from department and college.	○	○	○	○
Pursue training and stay current in science and/or science education.	○	○	○	○
Inform, educate, and highlight your efforts among your faculty colleagues and administrators.	○	○	○	○
Have a clear vision of and follow your professional interests.	○	○	○	○

64. What other advice you would offer to a beginning SFES?

About You ...

This section will ask demographic questions. As with the rest of the survey, any potentially identifying information in your responses will be kept strictly confidential and the survey data itself will be collected ANONYMOUSLY. We are interested in the set of responses as a whole, not a particular individual's responses.

About You ...

65. How would you characterize your gender identity?

◯ Woman ◯ Man ◯ Decline to state

◯ Other, please share how you identify.

66. What is your age?

◯ 20-29 ◯ 30-39 ◯ 40-49 ◯ 50-59 ◯ 60-69 ◯ 70+ ◯ Decline to state

67. With which group(s) do you most closely identify? Please mark all that apply.

- ☐ American Indian or Alaska Native
- ☐ Asian
- ☐ Black or African American
- ☐ Native Hawaiian or Other Pacific Islander
- ☐ White
- ☐ Decline to state
- ☐ Other (please describe)

68. With which ethnicity do you most closely identify?

◯ Hispanic or Latino

◯ Not Hispanic or Latino

◯ Decline to state

69. Are you in the first generation of your family to go to college?

◯ Yes

◯ No

70. Did you attend a community college as part of your undergraduate degree?

◯ Yes

◯ No

For the Future ...

71. If you have potential SFES colleagues in the CSU who you think should receive this survey, please list their names and campuses below. Your name WILL NOT be associated with survey invitations to these individuals.

For the Future ...

72. There have been discussions about holding a gathering of SFES from across the CSU to share insights, challenges, and successes, as well as to nucleate cross-campus collaborations.

If funds were available for travel, would you be interested in participating in this gathering?

- Yes
- No
- Maybe

For the Future ...

If you would like to be informed of publications that result from this study or plans for a gathering of SFES from across the CSU, please share your contact information below.

Please note, contact information entered on this page will be removed from your other survey responses. It will not be associated with any other information you have provided and will not be used for analysis.

If you would prefer to provide contact information external to this survey, please send your name, your institution's name, and your email address to csu.sfes.researchteam@gmail.com.

73. Please provide the following contact information.

Name:

Institution:

Email Address:

Thank You!

Thank you most sincerely for your time and thoughtfulness in participating in this study of SFES – Science Faculty with Education Specialties – in the California State University system.

If you would like to learn more about the SFES phenomenon both in the CSU and Nationally please visit: tinyurl.com/sfes-pubs
